# A case of hereditary metachronous bilateral triple-negative breast cancer that was highly sensitive to carboplatin

**DOI:** 10.1093/jscr/rjab018

**Published:** 2021-04-14

**Authors:** Rongrong Wu, Kayo Adachi, Yoichi Koyama, Kyoko Orimoto, Miki Okazaki, Mariko Asaoka, Saeko Teraoka, Ai Ueda, Kana Miyahara, Takahiko Kawate, Hiroshi Kaise, Kimito Yamada, Eichi Sato, Takashi Ishikawa

**Affiliations:** 1 Department of Breast Oncology and Surgery, Tokyo Medical University, Tokyo, Japan; 2 Department of Pathology, Tokyo Medical University, Tokyo, Japan

## Abstract

A 52-year-old woman with a strong family history of breast cancer was diagnosed as having triple-negative breast cancer (TNBC) in her right breast. Neoadjuvant chemotherapy (NAC; four cycles of epirubicin/cyclophosphamide/5-fluorouracil) was performed, followed by breast-conserving surgery and axillary lymph node dissection. Histopathological analysis of the surgical specimens demonstrated a few focal tumor cells remaining in the stroma, but not a pathological complete response (pCR). Weekly paclitaxel was subsequently added to the treatment regimen. A total of 17 months after the adjuvant treatments, TNBC recurred in her left breast with massive lymph node metastasis. Because of the early recurrence after standard treatment, NAC was administered together with carboplatin and paclitaxel. Histopathological analysis of the partially resected breast and axillary lymph nodes demonstrated a pCR. No recurrent disease was found 2 years after the second TNBC treatment. This case underlines the importance of platinum-based chemotherapy and prophylactic mastectomy for patients with BRCA dysfunction.

## INTRODUCTION

Triple-negative breast cancer (TNBC) accounts for 15–20% of all breast cancers, and the prognosis is generally unfavorable. However, clinical outcomes have been reported to be substantially improved after the achievement of a pathological complete response (pCR) in TNBC patients [1]. Thus, drug selection is important for the successful treatment of patients with TNBC.

Approximately 10% of TNBC patients have hereditary breast and ovarian cancer, and 30% of these patients have either BRCA1 or BRCA2 germline mutations. It has been reported that 60% of sporadic TNBC patients have similar functional alterations to those caused by BRCA1 mutations, and this group is called ‘BRCAness’ [2]. It was also reported that BRCA1 impaired cancers are sensitive to DNA damaging agents [3].

We report a case of a patient with hereditary TNBC, who experienced recurrence in her contralateral breast soon after standard treatment, which was successfully treated with a carboplatin regimen.

## CASE REPORT

**Figure 1:**
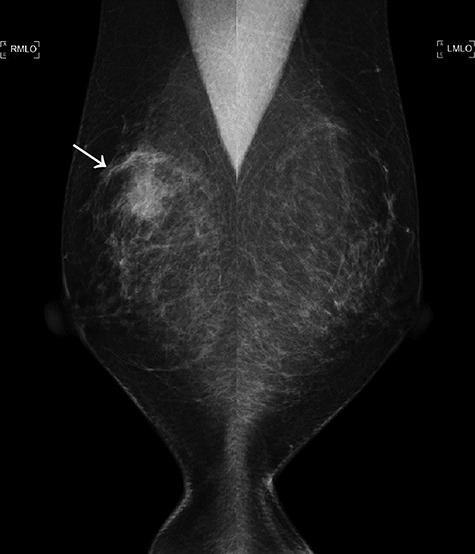
The first mammogram. The mammogram displaying a highly dense micro-serrated mass in the right upper lateral region (arrowhead).

**Figure 2:**
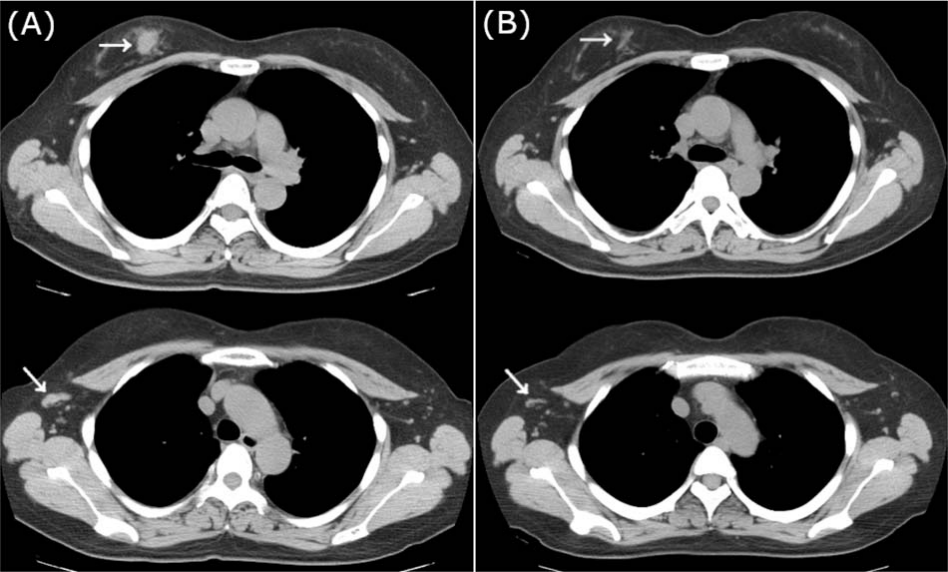
CT images of right breast tumor before and after treatment. (**A**) Chest CT displaying nodules in the right breast (top, arrowhead) and lymph node swelling in the right axilla (bottom, arrowhead); (**B**) after administration of NAC, the primary tumor (top, arrowhead) and axillary lymph nodes (bottom, arrowhead) displayed a clear reduction in size.

**Figure 3:**
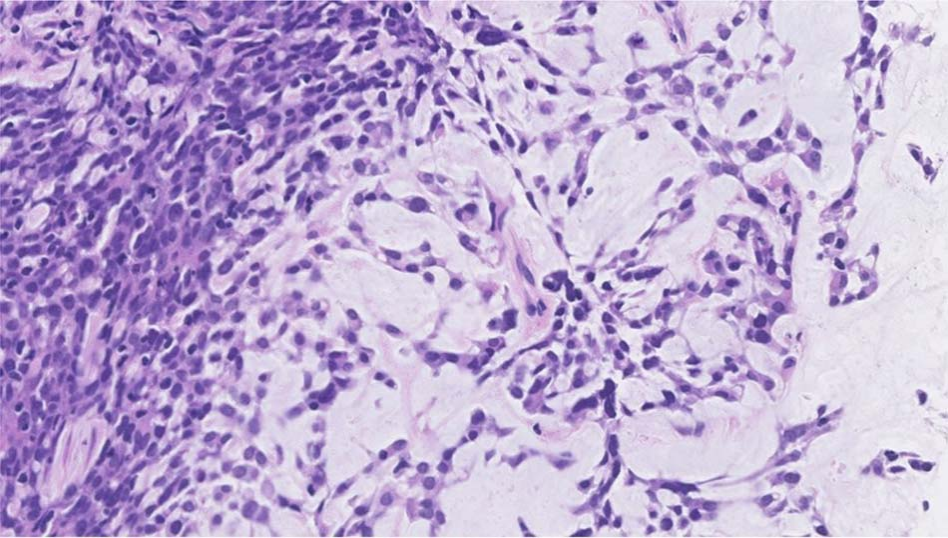
Histopathological features of right breast tumor. The biopsy specimen showed high-grade carcinoma cells arranged in solid sheet or thin trabecular structures directly producing matrix, suspected to be matrix-producing carcinoma (metaplastic carcinoma) (hematoxylin and eosin stain, ×400).

A 52-year-old woman with no medical history presented with an abnormality in her right breast that was detected on screening mammography. She has a strong family history of breast cancer, with her mother and twin sister being affected. Ultrasonography, mammography and computerized tomography displayed a 21 mm mass in her right breast (Figs 1 and 2A). A core-needle biopsy (CNB) demonstrated invasive carcinoma with features of matrix-producing carcinoma (Fig. 3). The clinical stage was cT2N0M0. Immunohistochemistry revealed that the tumor was negative for the estrogen receptor, progesterone receptor and human epidermal growth factor receptor 2 (HER2), with a Ki-67 score of 90%. Although BRCAnalysis^®^ test was recommended after genetic counseling, she refused it because of its lack of insurance coverage in Japan.

Neoadjuvant chemotherapy (NAC) was performed with four cycles of 5-fluorouracil (500 mg/m^2^), epirubicin (100 mg/m^2^) and cyclophosphamide (500 mg/m^2^) (FEC100 regimen), which showed a favorable response on imaging (Fig. 2B). She underwent surgery without taxane because she enrolled in a clinical trial (University Hospital Medical Information Network (UMIN) Clinical Trials Registry No.: R000029940) [4]. Postoperative treatment depends on the histopathological results. Based on the patient’s BRCAness score (0.6), she underwent breast-conserving surgery, and axillary lymph node (LN) dissection owing to a positive sentinel node biopsy. Pathological analysis demonstrated almost pCR in the breast and only a few degenerated carcinoma cells remaining in the stroma, with no metastatic carcinoma cells in the dissected LNs. Thus, she was sequentially administered 12 cycles of weekly paclitaxel and 50 Gy of radiotherapy.

**Figure 4:**
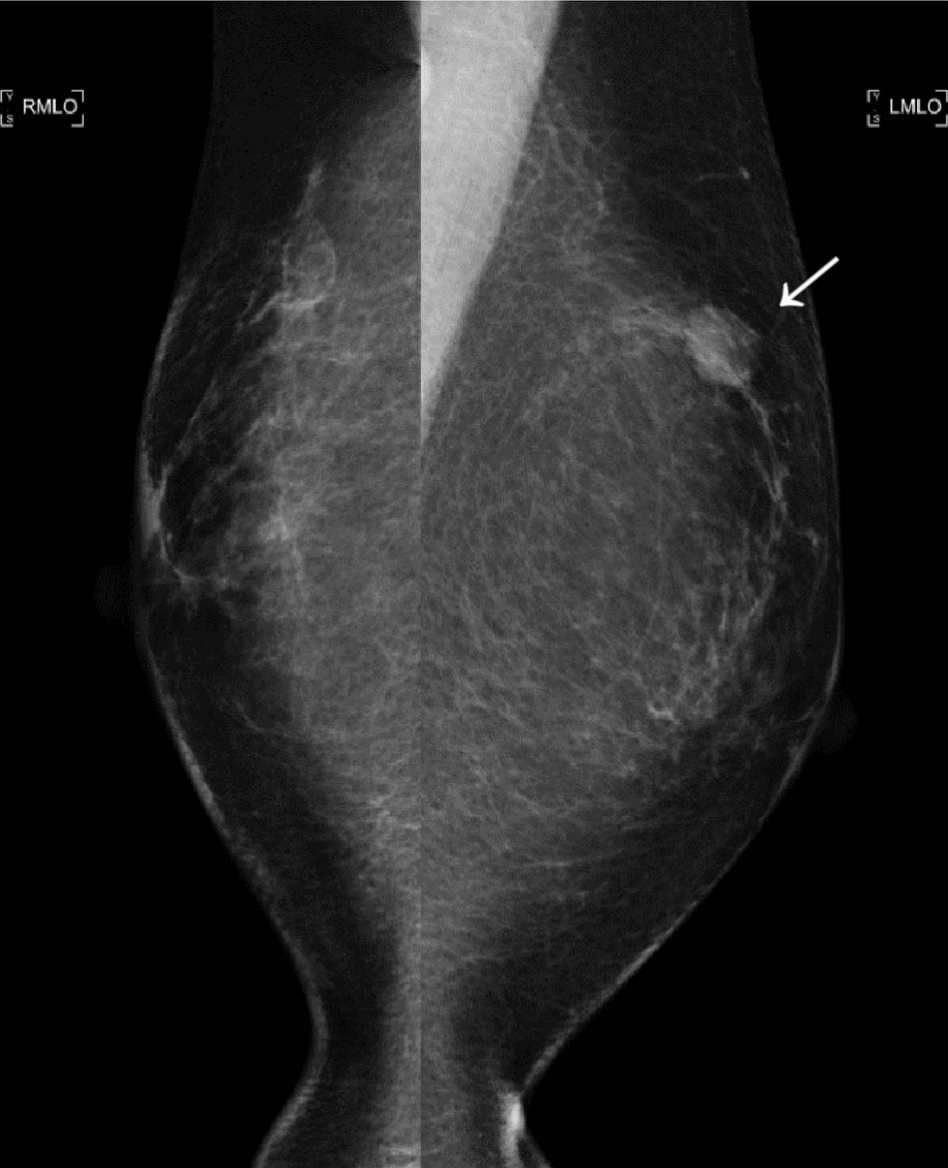
The second mammogram. The mammogram displaying a well-defined borderline high-density mass in the left upper lateral region (arrowhead).

**Figure 5:**
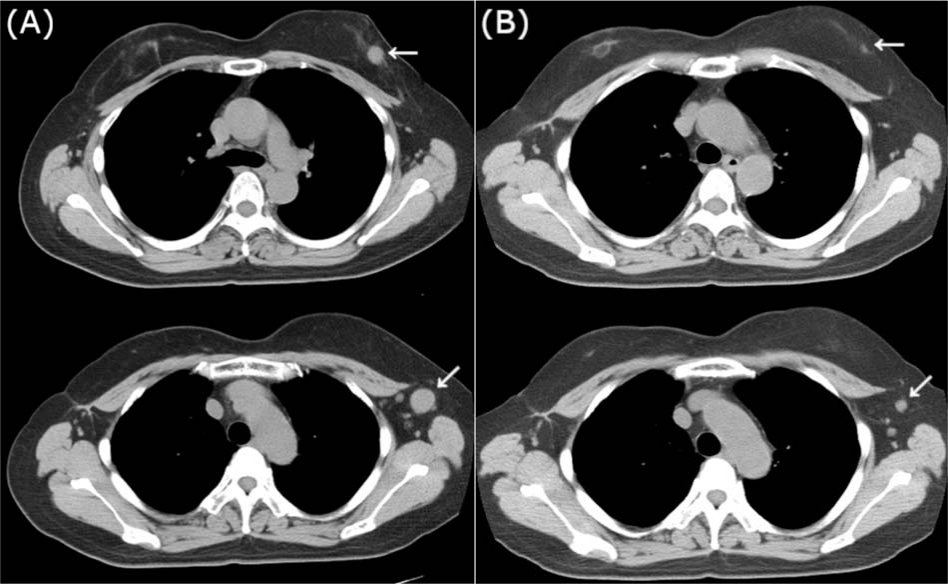
CT images of left breast tumor before and after treatment. (**A**) Chest CT displaying a nodule in the left breast (top, arrowhead) and lymph node swelling in the left axilla (bottom, arrowhead); (**B**) Tumors in both the left breast (top, arrowhead, arrowhead) and the axillary lymph node (bottom, arrowhead, arrowhead) shrank after carboplatin and paclitaxel administration.

**Figure 6:**
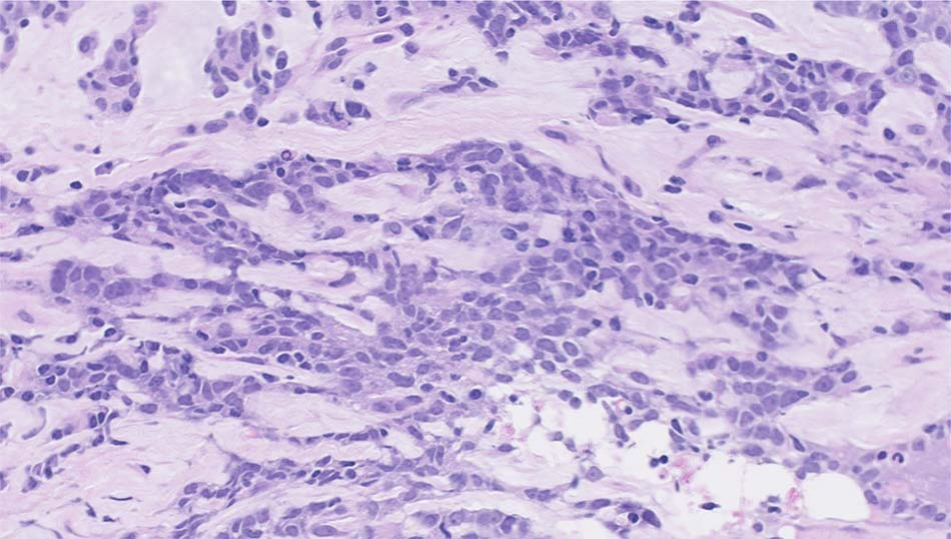
Histopathological features of left breast tumor. The biopsy specimen demonstrated invasive carcinoma with features of metaplastic carcinoma, i.e. without ductal structure, with tumor cell matrix-producing or some spindle cell characteristics (hematoxylin and eosin stain, ×400).

A total of 17 months after completing the adjuvant treatments, she noticed a mass in her left breast. Imaging analyses displayed a 15 mm mass in her left breast and multiple swollen axillary LNs (Figs 4 and 5A). Histopathological analysis of a CNB demonstrated metaplastic carcinoma (Fig. 6). The intrinsic subtype was TNBC with a Ki-67 score of 90%.

Because of the early onset of the opposite breast, we administered a combination of carboplatin (area under the concentration-time curve (AUC) 6.0) and paclitaxel (100 mg/m^2^) every 3 weeks for seven courses. Radiological analysis showed a favorable response (Fig. 5B), and she subsequently underwent breast-conserving surgery with axillary LN dissection. Pathological analysis demonstrated a pCR in the left breast and axillary LNs. She then underwent adjuvant radiotherapy in her left breast. There have been no signs of recurrence 2 years after the treatment.

## DISCUSSION

Considering this patient’s strong family history and bilateral TNBCs, her breast cancer is likely to be associated with BRCA1 mutations, although this was not genetically confirmed. The clinical course clearly showed that anthracycline was not sufficiently effective, whereas carboplatin was effective for her treatment.

Several studies showed the efficacy of platinum as an NAC in improving the pCR of TNBC patients. In the GeparSixto trial, the efficacy of adding carboplatin to NAC was analyzed for TNBC and HER2-positive breast cancers. The addition of carboplatin to a regimen of taxane, anthracycline and bevacizumab was shown to significantly increase the pCR rate in TNBC patients [5]. In the BrightNess trial, addition of the poly-ADP ribose polymerase (PARP) inhibitor veliparib plus carboplatin or carboplatin alone to standard NAC was analyzed in TNBC patients, including those with BRCA mutations. Unexpectedly, the addition of carboplatin improved the pCR rate, whereas the addition of veliparib was not beneficial, even in patients with BRCA mutations [6]. A meta-analysis including these two studies confirmed the efficacy of platinum in increasing the pCR rate for the treatment of TNBC patients [7]. These studies discussed whether platinum might be specifically crucial for treating BRCA-mutant or BRCA-deficient tumors in TNBC patients.

Recently, a randomized clinical study was conducted to compare the efficacy between anthracycline and cisplatin in BRCA-mutant patients receiving NAC. In contrast to previous studies, carboplatin did not show superiority in improving the pCR rate compared with anthracycline [8]. It was concluded that as anthracycline is also a DNA-damaging agent, BRCA-mutated breast cancers might simply be sensitive to any DNA-damaging agent, and not specifically to platinum agents.

It is still controversial whether platinum is essential for treating TNBC, particularly those with BRCA mutations. Regarding ovarian cancer, patients with BRCA mutations show favorable outcomes compared with sporadic patients, because of their sensitivity to platinum [9]. Comprehensive molecular analysis demonstrated a genetic similarity between serous ovarian carcinoma and basal-like breast cancer, which is closely associated with a deficiency of BRCA function [10]. This suggests that common therapeutic approaches will be effective for these two cancers, as platinum analogs are beneficial for BRCA function-deficient tumors.

In conclusion, we encountered a case of a patient with metachronous bilateral TNBCs with a strong family history. Our present case suggests that TNBC needs to be subdivided further for the selection of the most effective classical anticancer agents, particularly platinum-based regimens, and also for considering prophylactic mastectomy for patients with BRCA dysfunction.

## CONFLICT OF INTEREST STATEMENT

The authors declare no conflicts of interest associated with this manuscript.

## FUNDING

None.

## References

[1] Liedtke C, Mazouni C, Hess KR, André F, Tordai A, Mejia JA, et al. Response to neoadjuvant therapy and long-term survival in patients with triple-negative breast cancer. J Clin Oncol 2008;26:1275–81. 18250347 10.1200/JCO.2007.14.4147

[2] Lips EH, Mulder L, Oonk A, van der Kolk LE, Hogervorst FBL, Imholz ALT, et al. Triple-negative breast cancer: BRCAness and concordance of clinical features with BRCA1-mutation carriers. Br J Cancer 2013;108:2172–7. 23558900 10.1038/bjc.2013.144 PMC3670471

[3] Mori H, Kubo M, Nishimura R, Osako T, Arima N, Okumura Y, et al. BRCAness as a biomarker for predicting prognosis and response to anthracycline-based adjuvant chemotherapy for patients with triple-negative breast cancer. PLoS One 2016;11:e0167016. 27977696 10.1371/journal.pone.0167016 PMC5158199

[4] Teraoka S, Sato E, Narui K, Yamada A, Fujita T, Yamada K, et al. Neoadjuvant chemotherapy with anthracycline-based regimen for BRCAness tumors in triple-negative breast cancer. J Surg Res 2020;250:143–7. 32044511 10.1016/j.jss.2019.12.047

[5] von Minckwitz G, Schneeweiss A, Loibl S, Salat C, Denkert C, Rezai M, et al. Neoadjuvant carboplatin in patients with triple-negative and HER2-positive early breast cancer (GeparSixto; GBG 66): a randomised phase 2 trial. Lancet Oncol 2014;15:747–56. 24794243 10.1016/S1470-2045(14)70160-3

[6] Loibl S, O'Shaughnessy J, Untch M, Sikov WM, Rugo HS, McKee MD, et al. Addition of the PARP inhibitor veliparib plus carboplatin or carboplatin alone to standard neoadjuvant chemotherapy in triple-negative breast cancer (BrighTNess): a randomised, phase 3 trial. Lancet Oncol 2018;19:497–509. 29501363 10.1016/S1470-2045(18)30111-6

[7] Poggio F, Bruzzone M, Ceppi M, Ponde NF, La Valle G, Del Mastro L, et al. Platinum-based neoadjuvant chemotherapy in triple-negative breast cancer: a systematic review and meta-analysis. Ann Oncol 2018;29:1497–508. 29873695 10.1093/annonc/mdy127

[8] Tung N, Arun B, Hacker MR, Hofstatter E, Toppmeyer DL, Isakoff SJ, et al. TBCRC 031: randomized phase II study of neoadjuvant cisplatin versus doxorubicin-cyclophosphamide in germline BRCA carriers with HER2-negative breast cancer (the INFORM trial). J Clin Oncol 2020;38:1539–48. 32097092 10.1200/JCO.19.03292 PMC8462533

[9] Gallagher DJ, Konner JA, Bell-McGuinn KM, Bhatia J, Sabbatini P, Aghajanian CA, et al. Survival in epithelial ovarian cancer: a multivariate analysis incorporating BRCA mutation status and platinum sensitivity. Ann Oncol 2011;22:1127–32. 21084428 10.1093/annonc/mdq577 PMC6267858

[10] Yang D, Khan S, Sun Y, Hess K, Shmulevich I, Sood AK, et al. Association of BRCA1 and BRCA2 mutations with survival, chemotherapy sensitivity, and gene mutator phenotype in patients with ovarian cancer. JAMA 2011;306:1557–65. 21990299 10.1001/jama.2011.1456 PMC4159096

